# The role of eLearning in health management and leadership capacity building in health system: a systematic review

**DOI:** 10.1186/s12960-018-0305-9

**Published:** 2018-09-03

**Authors:** Lorainne Tudor Car, Bhone Myint Kyaw, Rifat Atun

**Affiliations:** 1 Family Medicine and Primary Care, Lee Kong Chian School of Medicine, Nanyang Technological University Singapore, 11 Mandalay Road, Singapore, 308232 Singapore; 2 Department of Primary Care and Public Health, School of Public Health, Imperial College London, Level 2, Faculty Building, South Kensington Campus, London, SW7 2AZ United Kingdom; 3 Department of Global Health and Population, Harvard T.H. Chan School of Public Health, 677 Huntington Avenue, Harvard, Boston, MA 02115 United States of America

## Abstract

**Background:**

Health leadership and management are essential for ensuring resilient health systems. Relevant training opportunities are often scarce, and the use of digital education could help address this gap. Our aim was to assess the effectiveness of eLearning for healthcare leadership and management capacity building.

**Methods:**

We performed a systematic review on the effectiveness of eLearning for health leadership and management training. We also reviewed literature on relevant competencies and training programmes. We conceptualise the role of health leadership and management capacity building in health system strengthening and explore the use of eLearning in this area.

**Results:**

No evidence was found on the effectiveness of eLearning for health leadership and management capacity guiding. Evidence on health leadership and management education effectiveness in general is scarce and descriptive and reports learning outcomes. We explore how various forms of eLearning can help meet specific requirements of health leadership and management training.

**Conclusions:**

Literature on the effectiveness of health leadership and management education is scarce. The use of eLearning could support this type of training by making it more accessible and tailored. Future research should be carried out in diverse settings, assume experimental designs, evaluate the use of information technology and report health system outcomes.

**Electronic supplementary material:**

The online version of this article (10.1186/s12960-018-0305-9) contains supplementary material, which is available to authorized users.

## Introduction

Health leadership and management is an essential component of health systems strengthening [1, 2]. It plays a key role in the provision of safe and effective healthcare and in ensuring health worker motivation and retention [3, 4]. In its new strategy on human resources for universal health coverage, the World Health Organization (WHO) highlights building capacity for effective public policy stewardship, leadership and governance as one of its four key objectives [5]. This is a timely and important call given the widespread shortage and inadequate education of health leadership and management cadre, particularly in low- and middle-income countries (LMIC) [1].

Health leadership serves to inspire, motivate and connect diverse stakeholders and organizations with the aim of achieving a shared vision. Health management focuses on administrative processes such as planning, budgeting, and organising, staffing, controlling and problem solving in relation to health services, resources and stakeholders. Health management can be further differentiated into top-level management in charge of policy, middle management with supervisory role and operations management responsible for health service delivery [6]. Health leadership and management roles, although in theory diverse, in reality often co-occur and are commonly assumed by clinicians whose training needs are largely unmet at both pre-service and in-service levels [7–9]. Healthcare professionals reported a number of challenges in relation to health leadership and management training, including lack of time, resources, access to experts or perceived relevance [10, 11]. By enabling accessible, relevant and timely training, eLearning (i.e. the use of digital technology for education) in health leadership and management could help address these barriers and improve health professionals’ knowledge, skills, attitudes, behaviours and satisfaction [12, 13].

Despite a diversity of commercial eLearning resources on health leadership and management available today, there is a lack of evidence on the use of digital technology for this type of training. Our objective was to assess the effectiveness of eLearning for health leadership and management capacity building on health system outcomes. We also sought to determine essential competencies as part of health leadership and management education as well as how they differ from competencies in other healthcare professionals’ roles. Finally, we explore health leadership and management educational trends in the literature and provide suggestions on the use of diverse eLearning modalities for health leadership and management capacity development.

## Method

### Systematic review of the literature and conceptualization of health leadership and management capacity building for health systems strengthening

We followed the Cochrane methodology and searched for randomised controlled trials (RCTs), cluster RCTs and quasi RCTs on the use of any type of eLearning modality for health management and leadership capacity building in all types of healthcare professionals. We used the following definition for health management: “Healthcare management is the profession that provides leadership and direction to organizations that deliver personal health services and to divisions, departments, units, or services within those organizations” [14]. We followed John Kotter’s definition of leadership as “a set of processes that creates organizations in the first place or adapts them to significantly changing circumstances. Leadership defines what the future should look like, aligns people with that vision, and inspires them to make it happen despite the obstacles” [15]. Capacity building was defined as an evidence-driven process of developing and strengthening the abilities of new as well as current workforce, organizations and systems [16]. ELearning was defined as the use of digital technology for education. We considered all eligible eLearning modalities including online learning, offline learning, mobile learning (mLearning), virtual reality environments, massive open online courses (MOOCs) and serious gaming. We were also interested in studies that used blended learning (i.e. a combination of digital and traditional learning methods) for health leadership and management education.

The primary outcomes of interest were health outcomes, financial risk protection and user satisfaction. We also searched for secondary outcomes of interest such as the attainment of health system objectives of improved equity, efficiency, effectiveness and responsiveness. We searched the following relevant databases of published and grey literature sources from 1990 onwards without language restrictions (Additional file 1): MEDLINE (Ovid), Embase (Elsevier), The Cochrane Central Register of Controlled Trials (CENTRAL) (Wiley), PsychINFO (Ovid), Educational Resource Information Centre (ERIC) (Ovid), Cumulative Index to Nursing and Allied Health Literature (CINAHL), Web of Science Core Collection (Thomson Reuters), ProQuest Dissertation and Theses Database, Google Scholar (first 500 references), Global Health (Ovid), Health Systems Evidence, PDQ-Evidence, Joint Bank-Fund Library (between the IMF and World Bank), the World Health Organization (WHO), USAID, Health Systems 20/20 and Management Sciences for Health. We searched the literature from 1990 onwards as the use of computers prior to that was uncommon and limited to basic tasks. The search strategy used in this review was designed, tested and refined by a team of researchers, content experts, librarians and information specialists.

### Identifying health leadership and management educational trends and competencies

We searched PubMed, Google and grey literature using a combination of major search terms such as healthcare, leadership, management, training, education and competencies and looked for literature reviews, systematic reviews, larger-scale surveys and opinion pieces. We were interested in studies in English language presenting health leadership and management competencies and educational programmes from all settings. We collated data on the healthcare context, type of healthcare professionals and the main categories of competencies that the paper is focused on. In addition, we were interested in the structure, organization and content of health leadership and management education from the literature.

## Findings and discussions

### Findings from the systematic review on eLearning for health leadership and management capacity building

**Fig. 1:**
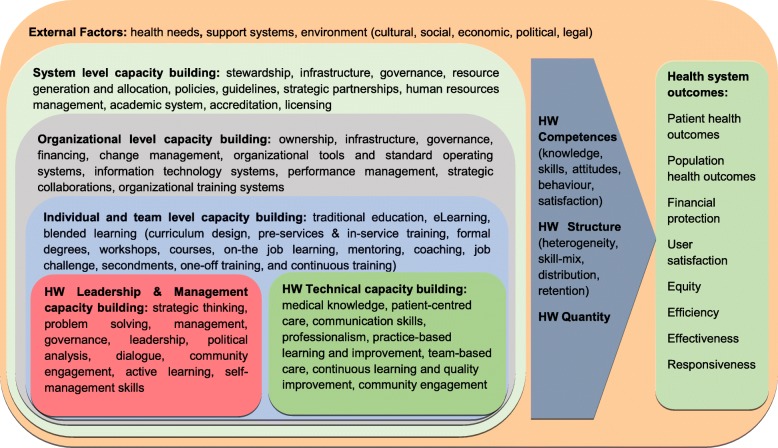
Conceptual framework for the use of eLearning for health workforce (HW) capacity building on health system outcomes (The listed activities in the framework are not meant to be exhaustive but to exemplify the relevant components)

Despite a sensitive and comprehensive search of a range of electronic databases and grey literature sources focused on healthcare management, we were unable to find any evidence on the effectiveness of digital education for health leadership and management capacity building. Here, we present the conceptual framework from our protocol that presents the role of health leadership and management capacity building (traditional and eLearning) in health system outcomes (Fig. 1) [17]. The framework builds on the Human Resources for Health Action Framework, the USAID Conceptual Model: Leading, Managing and Governing for Results and the President’s Emergency Plan for AIDS Relief (PEPFAR) Capacity Building Framework [18–20]. It does not aim to present a complete list of all relevant educational and contextual components but to convey the underlying principle. The framework contains three concentric levels at which capacity building can take place—system, organizational and individual level—and acknowledges external, contextual influence. It differentiates health leadership and management roles from technical (i.e. clinical practice-focused) roles in line with the WHO classification [21]. The immediate impact of capacity building activities is seen through improvements in healthcare workforce competencies, structure and quantity. These improvements in the long run translate into changes in health system outcomes, e.g. health outcomes, financial protection, user satisfaction, equity, efficiency, effectiveness and responsiveness. The observed impact and changes among different components within the frameworks are not unidirectional but interdependent. For example, effective capacity building could lead to improvements in health workforce and, consequently, in time lead to better health outcomes. Improved health outcomes could contribute to economic growth and beneficial context (i.e. external factors) which in turn can further improve health workforce capacity building activities.

### Health leadership and management competencies: what do we mean exactly and is it context-dependent

We found a number of existing literature reviews on healthcare leadership competencies [22–28]. They mostly stemmed from high-income countries (HIC) and focused on the development of leadership competencies in nursing staff and physicians. We also found several national frameworks on healthcare leadership competencies including the Australian LEADS framework [29], Canadian leadership (LEADS) framework [30] and the United Kingdom’s NHS Healthcare Leadership Model [31].

Based on the preliminary analysis of the findings, we noticed substantial overlap in competencies across these reviews with most competencies belonging to one of the following categories:

- Management of resources. This group of competencies is related to the successful management of resources such as staffing, financing, information, digital technology and physical resources.
- Management of processes. The processes are defined as a combination of steps and activities that create some output or result. This set of competencies include competencies relating to service organization, quality improvement, data collection, change management, vision setting, etc.
- Management of relationships. These competencies help achieve successful and productive relationships with colleagues and healthcare service consumers through teamwork, supervision, performance management, work assignment, monitoring, mentoring, networking, organizational culture, etc.
- Self-management. This set of competencies relates to personal development through competencies relating to time-management, stress-management, productivity, communication skills, emotional intelligence, leading ethically and with integrity, etc.
- Management within a context. This set of competencies relates to familiarity with the environment, i.e. organizational culture, community needs, reimbursement type, competition, policies, regulations, laws, organizational structure as well as systems thinking, political prowess and industry knowledge.

Our review revealed a lack of conceptual clarity as to what healthcare leadership is and what it is for. While performing our systematic review, we encountered several studies that focused on leadership training in the form of non-technical skills development (e.g. teamwork, communication) in clinicians as part of direct patient care. Others focused on the development of research or educational skills, which did not seem closely related to health leadership and management. We have also noticed that, in addition to clinical practice, leadership training in the literature was often mentioned in the context of other healthcare professionals’ responsibilities such as medical education, academia, public (and global health) and patient safety. In trying to delineate how health leadership and management roles differ from other healthcare professionals’ roles, we followed the WHO health system strengthening framework, which includes six additional building blocks: service delivery, health workforce, health information systems, access to essential medicines and financing. Leadership (and health information systems) is considered a cross-cutting theme which enables overall policy and regulation of the other system blocks. Correspondingly, we define health leadership primarily with regard to its managerial scope, focusing on health service delivery and organization and through it, improvement of the health of communities affiliated to that particular health service.

Yet healthcare professionals may take on leadership roles in a range of other areas such as clinical practice, medical education, academic research, innovation and public health. The boundaries among these various healthcare leadership roles are in reality often malleable. Furthermore, some healthcare leadership and management competencies, particularly those focused on self-management and relationship management, are also relevant for a range of healthcare leadership roles. Other sets of competencies such as management of resources and processes are more relevant to managerial responsibilities. In conceptualising the health leadership role as having a management focus, the greatest overlap arises in relation to public health and global health leadership, both being interdisciplinary fields striving to improve the health of the community. However, the organization of health service delivery within public health is aligned with its primary goal of “preventing disease, prolonging life and promoting health”. Healthcare management is therefore broader in scope as it can encompass organization of health services supporting diagnosis, treatment, rehabilitation, palliative care, etc.

With the exponential growth in information and limited time at hand, clinicians need customised education, tailored according to their needs and preferences. Rather than adopting existing broad competency frameworks, the developers of health management and leadership educational resources should seek to understand the requirements of particular positions and identify essential competencies taking into consideration:

- Setting. The essential competencies that need to be acquired as part of leadership and management in LMIC can be different to those relevant for HIC settings. The difference in manpower, resources, education and safety-netting augments the importance of health leadership and management staff in LMIC counties. Furthermore, health leadership and management roles can differ in a rural setting compared to an urban setting due to differences in access, burden of disease and cultural context. Another factor that can affect the most pertinent choice of competencies is the degree of involvement of non-clinical staff in healthcare management in a certain healthcare system. The rapport and division of responsibilities between non-clinical and clinical managers can have a major impact on the choice of most relevant competencies.
- Type of organization. The type of financing (public vs private) as well as size of organization (team, department, polyclinic or hospital) can have an important impact on the most pertinent healthcare leadership and management competencies. Furthermore, healthcare management in the primary care setting, mostly comprising of smaller types of healthcare organizations collaborating closely with community services, differs to that in hospitals given its size, complexity and diversity of stakeholders.
- Type of healthcare professional. The literature mostly focuses on the health leadership training and competencies of physicians, nurses and public health specialists. There is a scope for more data on health leadership and management education of other healthcare professional groups such as dentists, pharmacists and physiotherapists. The choice of key competencies may differ in relation to the career level, i.e. among undergraduates, early-career, mid-career and senior healthcare professionals.

### Trends in health leadership and management education research

We were interested to learn about the structure, organization and content of health leadership and management education in general from the literature. We retrieved several (systematic) reviews examining different forms of education for health leadership and management in a variety of settings and at different healthcare professional career levels [22, 23, 27, 32–36]. The literature primarily stemmed from HIC such as the United States of America, the United Kingdom, Canada and Australia was mostly descriptive, focused on one type of professional and took place in academic settings. It reported on basic learning outcomes (such as knowledge and attitudes) and did not report on health system outcomes. Training approaches spanned didactic lectures, discussions, mentoring, experiential learning and group work while eLearning and blended (i.e. combination of digital and traditional learning methods) programmes were rare. The reported training programmes lasted from one term to 1 year and differed in terms of providers, pedagogical approach, content, duration and intensity. A review on leadership education in the United Kingdom highlighted organizational barriers such as lack of protected time, funding, space, access and the distinctiveness of the United Kingdom health systems in having the lowest proportions of clinically qualified managers [27]. A LMIC-focused review reported that clinical experience and competence were prerequisites for managerial positions and training was mostly in the form of on-the-job learning or short training courses [32]. Some grey literature sources, such as the Management Science for Health website, reported on individual programmes implemented in LMIC and offered guidance on how to develop health leadership and management capacity in resource-constrained settings [37–39].

There is very little evidence of any design on digital education for health leadership and management with only a few rare and diverse case studies. We found several studies from LMIC that reported using blended learning, i.e. a combination of online modules and face-to-face, collaborative, project-based learning. These included reports on a co-learning partnership programme in Global Health between African and American universities called Afya Bora [40–42]. This 1-year fellowship aims to provide inter-professional leadership training through blended learning in six African countries on design, implementation, scale-up, evaluation and leadership of HIV and other health programmes. Next, we also found reports of a collaborative, blended and project-based programme from 12 African countries for human resources (HR) managers [43]. This 13-week programme, focused on HR issues and solutions, included an online module, regular team meetings and a capstone project. Upon completion, participants reported the successful application of learned concepts and improved collaboration within organizational teams. One study reported on the Virtual Course on Primary Health Care-based Pharmaceutical Services implemented in the Latin American and the Caribbean regions with the aim of improving the reach of geographically dispersed primary care-based pharmacy managers [44]. The challenges encountered included participants’ poor access to the internet and limited applicability to the real world.

We also found two studies on health leadership and management that used digital education conducted in HIC. One presented a 12-week online learning community via a wiki (i.e. website or database developed collaboratively by a community of users, allowing users to add and edit content) for nurse educators from three Canadian provinces [45]. The aim was to share and learn about exemplary leadership practices, and participants reported significantly improved leadership practices after participation. Another example from Holland reported on the use of the flipped classroom approach as part of a leadership training module for medical residents on healthcare law and medical errors [46]. A flipped classroom approach was chosen with a view of promoting better participants’ engagement and higher cognitive skills development. Participants had access to online educational resources (e.g., e-journals and e-books) and open-access videos before taking part in two classroom-based sessions. Authors noted that the module was well-received but also credited educators’ substantial investment of time and effort in its development.

While the literature on the use of digital education for healthcare leadership and management is scarce, there are a range of existing relevant educational resources that employ eLearning, available on the market including:

- Numerous distance and blended degrees worldwide [47, 48]
- Massive open online courses available by major MOOC providers [49–51]
- A variety of resources that are primarily focused on health leadership and management in resource constrained settings offered by organizations such as the WHO and the World Bank [52–54]
- Online modules accompanying national health leadership competency frameworks. In the United Kingdom, the NHS Leadership Academy offers a variety of leadership programmes for clinicians at different entry levels alongside corresponding eLearning resources such as “Leadership for clinicians” [55, 56].

### Potential benefits of using eLearning for health leadership and management training

This contrast between the scarce evidence base and an abundance of digital education opportunities prompted us to explore the potential benefits of the use of eLearning in relation to healthcare management and leadership training. Below, we list the most important attributes that successful health leadership and management education need to fulfil and how eLearning can help to meet these requirements:

- Accessible. Healthcare professionals are busy, and their managerial role is often an add-on to their existing roles and responsibilities. Therefore, it is important that potential barriers (e.g. lack of time, distance, lack of Internet connection) that may exist in relation to education are removed. This could be achieved with the use of mLearning, i.e. mobile device-delivered educational resources as it allows just-in-time, asynchronous and self-paced learning, with some educational programmes available offline.
- Relevant. Traditional leadership training can be formal, untailored and outdated. ELearning resources can be more readily modernised and customised to the participants’ needs. Furthermore, the use of eLearning data analytics could enable more focused and timely learning. Sharing of updates can be achieved through the use of emails, text messages, e-articles, infographics, podcasts, etc. as well as more comprehensive formats such as refresher online courses.
- Engaging. Attitude toward the learning process is crucial for its success. In order to capture busy healthcare professionals’ attention, there is a need to make the learning process as engaging as possible. The educational content can be made appealing with the appropriate use of multimedia, videos, reminders, immersion or gamification. In addition, eLearning also supports collaborative learning, an essential part of health leadership and management training, with the use of online forums, wikis, virtual classrooms, e-conferencing, chats and information sharing.
- Scalable. Reaching geographically dispersed healthcare professionals was a motivation for the use of blended learning approach in the presented case studies. ELearning modalities that lend themselves particularly well toward this goal include MOOCs, co-learning partnerships between LMIC and HIC institutions, podcasts, text-messaging, emails, etc. Customization of content can also be achieved even in large-scale educational programmes with the use of eLearning forms such as coaching and project-based learning.

Health leadership and management competencies vary ranging from knowledge on regulations, laws, policies, organizational structure and process to learning soft skills such as self-management and management of relationships. This diversity of competencies in health leadership and management education translates to different educational approaches that can be successfully supported with eLearning as presented below:

- Sharing of information. At times, health leaders and managers are required solely to familiarise themselves with pertinent information which can be achieved through the use of a range of eLearning forms such as online modules, bite-size learning, e-articles, online forums, infographics and videos.
- Experiential learning through simulation. Some health leadership and management competencies require the development of cognitive and technical skills, which is best achieved with the use of guided discovery. ELearning modalities such as virtual reality environments, case-based learning and project-based learning which enable immersion, interactivity, feedback and tracking may be particularly helpful for this type of training.
- Supporting collaboration. Collaboration and teamwork competencies are a must in health leadership and management. ELearning offers numerous opportunities for connecting with colleagues and creating a community of like-minded healthcare professionals that share ideas and collaborate, e.g. through the use of online forums, chats, wikis and e-conferencing.
- Providing performance support. The decision-making process is complex, and digital technology offers a solution in the form of just-in-time access information through the use of cognitive or job-aids such as checklists and e-manuals.

ELearning also allows easier catering to healthcare professionals at various career stages enabling different forms of education. Blended modules with longer duration of the interventions may be more appropriate for undergraduate and early career health leadership and management training. Conversely, shorter, refresher-type and focused educational resources may be more suitable for more experienced healthcare professionals.

### Potential disadvantages of using eLearning for health leadership and management training

Coupled with this variety of training opportunities that eLearning offers, there are several potential challenges associated with the use of this type of education for health leadership and management training. At times, eLearning may not be the most optimal format of training. For instance, experienced health leaders and managers may benefit more from in-person sharing, discussion and mentoring. Also, certain skills pertaining to the management of relationships or self-management may be best taught face-to-face or in a blended format. Furthermore, eLearning content needs to be of high-quality, up-to-date and contextually appropriate in order to be effective. Digital versions of lecture notes and power-point presentations may not be an appropriate training resource for busy clinicians, and more engaging eLearning resources may be necessary. However, more elaborate eLearning resources require support staff that can assist in the creation and tailoring of relevant eLearning materials. The development and maintenance of eLearning may also be prohibitively expensive in certain settings. Despite the relentless pace of innovation in digital technology, it is important to match the eLearning interventions with the needs of the healthcare professionals and the available resources instead of pursuing the implementation of the latest eLearning trend. ELearning may also be inaccessible at times due to a lack of internet connection or equipment. Poor digital literacy among learners can potentially reduce usefulness and effectiveness of eLearning. Finally, healthcare professionals may lack protected time for this type of training leading to low uptake and high attrition rates. The use of eLearning for healthcare professionals’ training on health leadership and management evidently calls for a carefully planned strategy that will aim to address these various challenges [13, 57, 58].

### Strengths, limitations and future research priorities

The strengths of this manuscript include a very thorough search of a range of electronic databases and grey literature sources for studies assessing the effectiveness of eLearning in health leadership and management education. However, our exploration of health leadership and management competencies and trends in literature is based on a non-systematic review and we may have missed some relevant studies. Yet, instead of achieving high sensitivity of search, our aim was primarily to explore and conceptualise the role of eLearning in health leadership and management training by using a representative sample of studies.

There is a need to close the current evidence gap by performing methodologically robust research that would test outlined premises and help determine what works, for whom, at what cost and in which circumstances. The application of realistic methods in this area could be one of the potential solutions to research questions in future. We propose the use of high-quality experimental studies with organization-level assessment, pragmatic approach and LMIC settings as well as the inclusion of relevant health system outcomes, cost analysis and longitudinal collection of data. Future research should aim to explore the use of various eLearning modalities to support health leadership and management training among diverse healthcare professional groups.

## Conclusion

While examples of eLearning for health leadership and management education worldwide are plentiful, a relevant evidence-base is missing. The literature on health leadership and management education, in general, is descriptive and reports didactic programmes, from HIC, targets physicians and nurses and reports exclusively educational outcomes. This is coupled with ambiguity in relation to the scope of healthcare leadership role(s) and diverse contexts in which health leadership and management takes place. In this paper, we explore and conceptualise what health leadership and management is and how digital education can help meet educational requirements and learning aims that are specific to health leadership and management capacity building. By enabling accessible, easily updated, scalable and engaging training, eLearning has the potential to transform health leadership and management education. ELearning has the potential to cater to various training needs by facilitating information sharing, experiential learning, collaboration and just-in-time support. Future studies need to robustly evaluate the effectiveness of eLearning interventions for health leadership and management capacity building.

## Additional file

Additional file 1: MEDLINE (OVID) Search Strategy and the list of grey literature used. (DOCX 20377 kb)
